# Longitudinal weight and plantar pressure distribution while standing after tibial or malleolar fractures in patients with or without fracture union

**DOI:** 10.1038/s41598-024-75732-3

**Published:** 2024-10-24

**Authors:** Elke Warmerdam, Sonja Baumgartner, Tim Pohlemann, Bergita Ganse

**Affiliations:** 1 Werner Siemens-Endowed Chair for Innovative Implant Development (Fracture Healing), Departments and Institutes of Surgery, Saarland University, Homburg, Germany; 2 Department of Trauma, Hand and Reconstructive Surgery, Departments and Institutes of Surgery, Saarland University, Homburg, Germany

**Keywords:** Fracture healing, Instrumented insoles, Nonunion, Pedography, Postoperative monitoring, Static sway, Predictive markers, Fracture repair

## Abstract

Fracture healing is usually monitored by clinical impressions and radiographs. Objective and easy methods for assessing fracture healing without radiation would be beneficial. The aim of this study was to analyse whether weight and plantar pressure while standing can be used to monitor healing of tibial or malleolar fractures and whether these parameters can discriminate between patients with and without union. Thirteen patients were longitudinally assessed during each postoperative clinical visit, of whom two developed a nonunion. Eleven matched healthy controls were assessed once. Additionally, five patients already experiencing nonunion were assessed once at the time of their nonunion diagnosis. All participants performed a standing task for ten seconds with pressure-sensing insoles. Greatest improvements were detected throughout the first three months in patients with union. However, six months after surgery, more than half of the parameters were still significantly different from those of the controls. The weight and pressure distributions did not differ between patients with or without union six months after surgery. A standing task can be used to monitor improvements in weight and pressure distribution throughout the healing process of tibial or malleolar fractures, but lacks potential to discriminate between patients with or without fracture union.

## Introduction

The recovery process after bone fractures is usually monitored by clinical impressions combined with infrequent radiographs. Radiography has the disadvantage of X-ray exposure and only shows bone healing with a time delay^1^. In an attempt to develop new monitoring techniques for rehabilitation after injuries and surgeries, numerous wearable and gait analysis-based technologies have recently been tested for their predictive and clinical value^2,3^. When screening possible technologies, their applicability in terms of complexity and time consumption in a clinical context is a crucial factor. The weight and plantar pressure distributions when standing are easily assessable, but have not yet been studied as tools for monitoring the progress of recovery after tibial or malleolar fractures.

After a lower leg fracture, the injured leg is usually subjected to decreased loading due to pain and instructions for partial weight bearing^4,5^. Loading is expected to increase again during the healing process in patients who achieve union^6^. According to studies analysing gait, the plantar pressure shifts posteriorly during the healing of lower leg fractures^7^, and one to four years after injury, the pressure is located more laterally than that on the uninjured side^8–11^. Additionally, patients with degeneration of the ankle joint following a malleolar fracture exhibit decreased loading of the lateral forefoot when walking^10^. A patient with nonunion of a tibial shaft fracture showed an anterior shift in the centre of pressure (COP) during walking compared to patients with union, who showed a posterior shift in the COP^7^. Thus, plantar pressure data could potentially be used to detect nonunion. Nonetheless, there is no information available for tasks other than walking after tibial or malleolar fractures.

In daily life, approximately 30% of waking hours are usually spent standing^12,13^, making it a relevant position to monitor. Injuries or disorders may lead to changes in standing posture, such as asymmetries in weight and pressure distribution^14,15^. With asymmetric weight bearing, the sway length increases^16,17^. Moreover, people with a history of falls are known to have more sway than those without a history of falls^18^. Therefore, it is important that patients return to a symmetric stance posture.

For these reasons, we hypothesized that longitudinal changes in weight and plantar pressure distribution occur after tibial or malleolar fractures. The weight distribution between the injured and uninjured sides and the plantar pressure distribution on the injured side were hypothesized to improve throughout the healing process and to return to the levels observed in healthy controls within six months. Additionally, it was hypothesized that patients without fracture union would bear less weight and distribute their weight differently on their injured side than patients with union.

## Methods

Ethics approval was obtained from the Institutional Review Board of Saarland Medical Board (application number 30/21). Written informed consent was obtained before the start of the measurements. This study was performed according to the Declaration of Helsinki.

### Participants

Particpants were recruited between February 2022 and December 2023. Patients with a recent tibial or malleolar fracture were included in the longitudinal observational study. Additionally, patients with a diagnosed tibial fracture nonunion were included with a single measurement when they presented to our nonunion outpatient clinic. Nonunion was defined as a lack of callus bridging on radiographs approximately 6 months after surgery^19^. The exclusion criteria for both patient groups were age younger than 18 years, mobility limitations before the fracture, other injuries or disorders that affect standing, inability to give consent, and pregnancy. A healthy control group was age-matched to the patients with union, and identical measurements were performed. The exclusion criteria for the control group were age younger than 18 years, injuries or disorders that impact movement, inability to give consent, and pregnancy.

### Data collection

The first measurement of the patients who were monitored longitudinally was conducted in the hospital ward within the first days after surgery. The second, third and fourth measurements took place during scheduled outpatient visits in Saarland University Hospital at approximately six weeks, three months, and 6 months after surgery, respectively. Measurements were obtained once from the patients that were referred from peripheral healthcare providers to the nonunion outpatient clinic in this University Hospital. These patients were measured after their first visit at which the nonunion was confirmed, which was approximately 6 months after the fracture^19^. Measurements were obtained from those in the control group once.

The pressure distribution was assessed with pressure-sensing insoles containing 16 pressure sensors (OpenGO insoles, Moticon GmbH, Munich, Germany). These insoles have been validated against a force plate and had ICC values ranging between 0.94 and 0.99 for walking^20^. The insoles were matched for shoe size and were calibrated to the participants’ body weight by an individual calibration procedure according to the insole software, which was run on a tablet. The software was also used to record and save data, which were recorded at 100 Hz.

Participants were asked to stand upright in their preferred stance with their feet hip-width apart and to hold this position for 10 s. Those in the patient group were allowed to perform the task with crutches according to their partial weight-bearing instructions and how they felt comfortable. In patients with a malleolar fracture, the first two measurements were obtained while the patient was wearing a walker boot (VACOped, OPED, Valley, Germany), as this boot was part of the treatment protocol.

### Data analysis

**Fig. 1:**
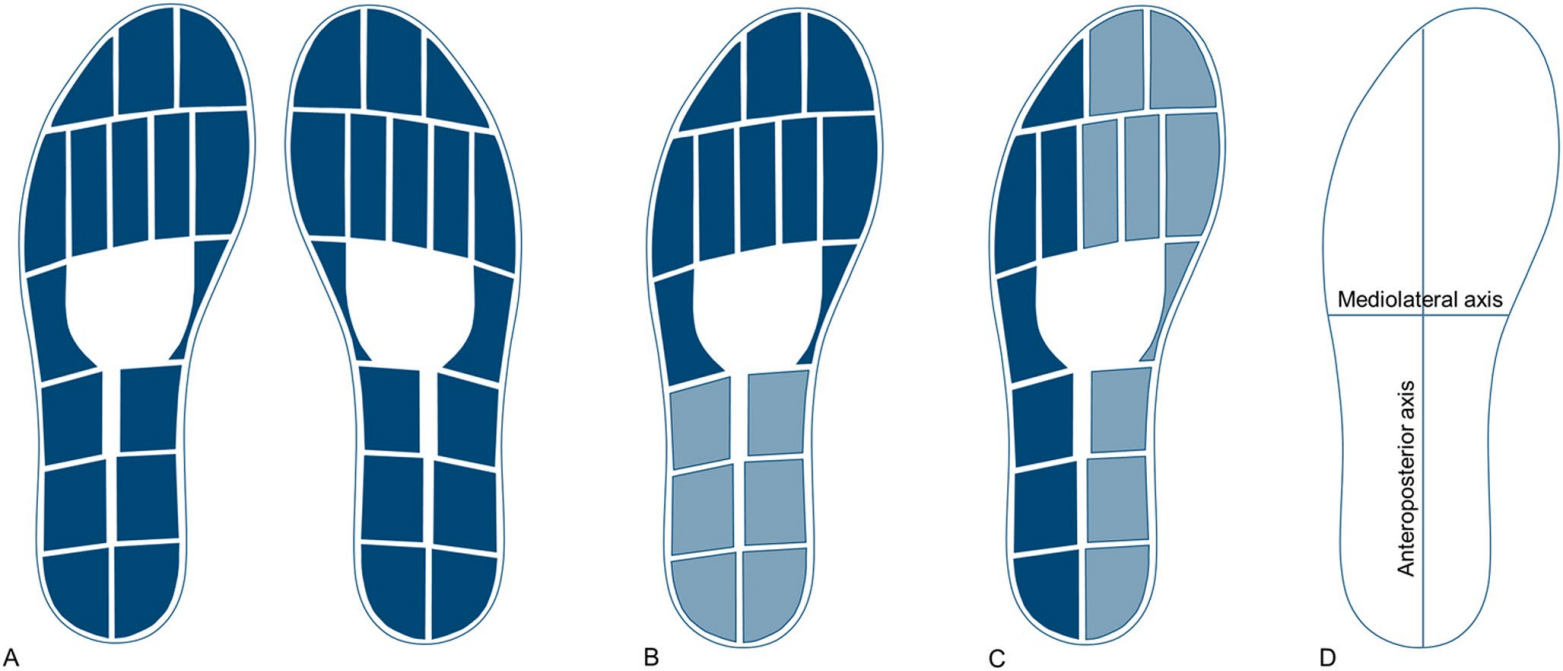
Layout of the sensors in the insole and the combination of sensors for each parameter. (**A**) All sensor data were used to calculate the weight distribution per side. (**B**) The combination of sensors for forefoot and hindfoot pressure. (**C**) The combination of sensors for lateral and medial pressure. (**D**) The direction of the axis for the centre of pressure parameters.

Data were filtered with a fourth-order Butterworth filter with a cut-off frequency of six Hz. The total vertical force data were normalized to the body weight of each participant. The average force over the ten seconds was calculated to quantify how participants distributed their weight. To analyse and compare the pressure on different parts of the feet, data from several pressure sensors were combined in different variations (Fig. 1). The data from each pressure sensor are expressed in N/cm^2^. These values were computed as the percentage of body weight per cm^2^ for comparison. For the pressure underneath different parts of the feet, single-sensor data were summed and then divided by the number of sensors to calculate the average pressure as the percentage of body weight per cm^2^ over the ten seconds. COP data were calculated by the software of the pressure-sensing insoles (OpenGo, Moticon GmbH, Munich, Germany). The COP sway length was calculated by summing the distances covered and was calculated in the anteroposterior (AP) and mediolateral (ML) directions, as well as combined with the total sway length^21^. The COP range was defined as the difference between the minimum and maximum COP positions and was calculated in both the AP and ML directions. Additionally, the asymmetry between the injured and uninjured side of the before mentioned parameters was calculated:

$$\:Asymmetry=\:\frac{(uninjured\:side-injured\:side)}{(0.5*(uninjured\:side+injured\:side))}*100$$

### Statistical analysis

Normality was tested with Shapiro‒Wilk tests. Differences in demographics between the patients with fracture union and healthy controls, as well as between patients with and without fracture union, were analysed by independent t-tests. Longitudinal changes in the patient group with union were analysed by repeated-measures ANOVA. When sphericity was violated, a Greenhouse‒Geisser correction was applied for the repeated-measures ANOVA. Post hoc tests were performed according to the Holm method. The data of patients with union at six months after surgery were compared with those of healthy controls by independent t-tests to determine whether long-term abnormalities were still present. The data of patients with and without fracture union approximately six months after injury were compared to determine whether stance analysis has the potential to detect nonunion. Mann‒Whitney U tests were used instead of independent t-tests for data that were not normally distributed. Significance was assumed at *p* < 0.05 for all tests.

## Results

**Fig. 2:**
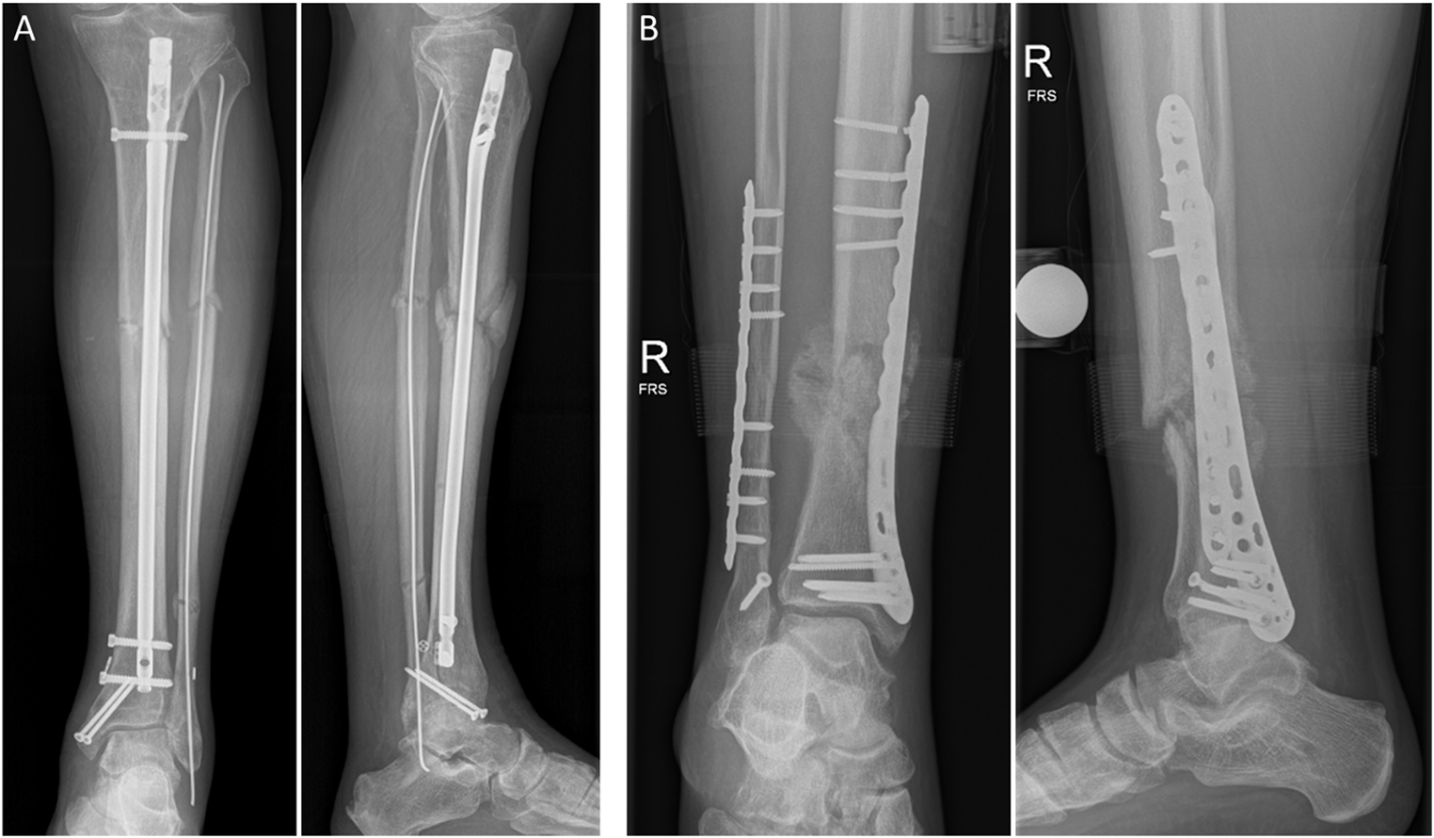
Radiographs of two patients with nonunion. (**A**) A 71-year-old man 140 days after surgery. (**B**) A 28-year-old man 125 days after surgery. Note the untreated syndesmotic diastasis that has likely contributed to the nonunion development.

Thirteen patients with tibial or malleolar fractures were assessed longitudinally. This group consisted of five patients with a proximal tibial fracture, four patients with a tibial shaft fracture and four patients with a malleolar fracture. Two of the tibial shaft fracture patients were later diagnosed with nonunion. One of these patients, who was treated with a tibial nail, had diabetes and smoked, which are known risk factors for fracture healing-related complications (Fig. 2A)^22^. The other patient was treated with an angle-stable plate on the tibia, and due to excessive loading, the proximal screws broke (Fig. 2B). His last assessment took place before the revision surgery. Besides the two longitudinal nonunion patients, five additional nonunion patients that had initially been treated elsewhere and were then referred to the nonunion outpatient clinic of our hospital were enrolled in this study and measured once. For comparison, only the last measurement of each of the two longitudinal nonunion patients was used, because this matched the measurement time point of the patients from the outpatient clinic. Accordingly, a total of seven patients (one proximal tibia and six tibial shaft fractures) were included in the nonunion group that was assessed on average 186 days after surgery.

**Fig. 3:**
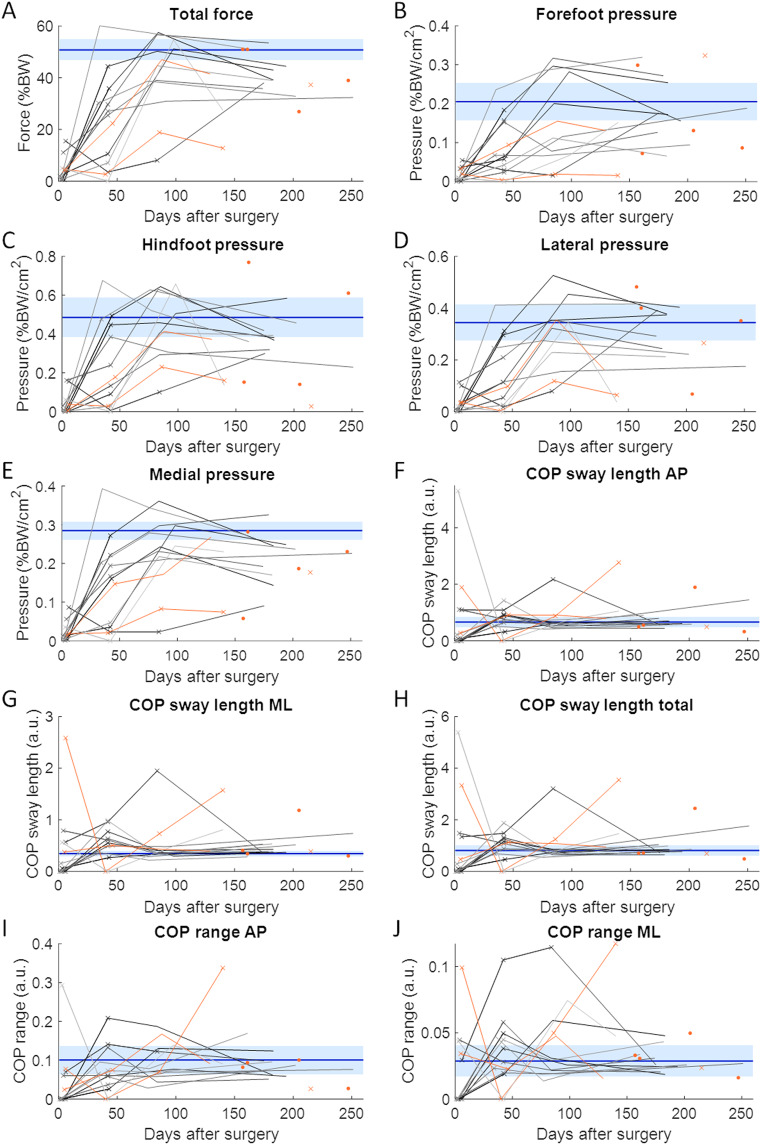
Average weight and plantar pressure distribution parameters. The horizontal blue solid line and the light blue shaded area indicate the average and the 95% confidence intervals of the control group, respectively. Each longitudinally assessed patient is represented by a different line. The datapoints marked by a cross indicate that the patient performed the assessment with crutches. The datapoints and lines in orange present the patients experiencing nonunion. AP = anteroposterior; a.u. = arbitrary units; BW = body weight; COP = centre of pressure; ML = mediolateral.

**Fig. 4:**
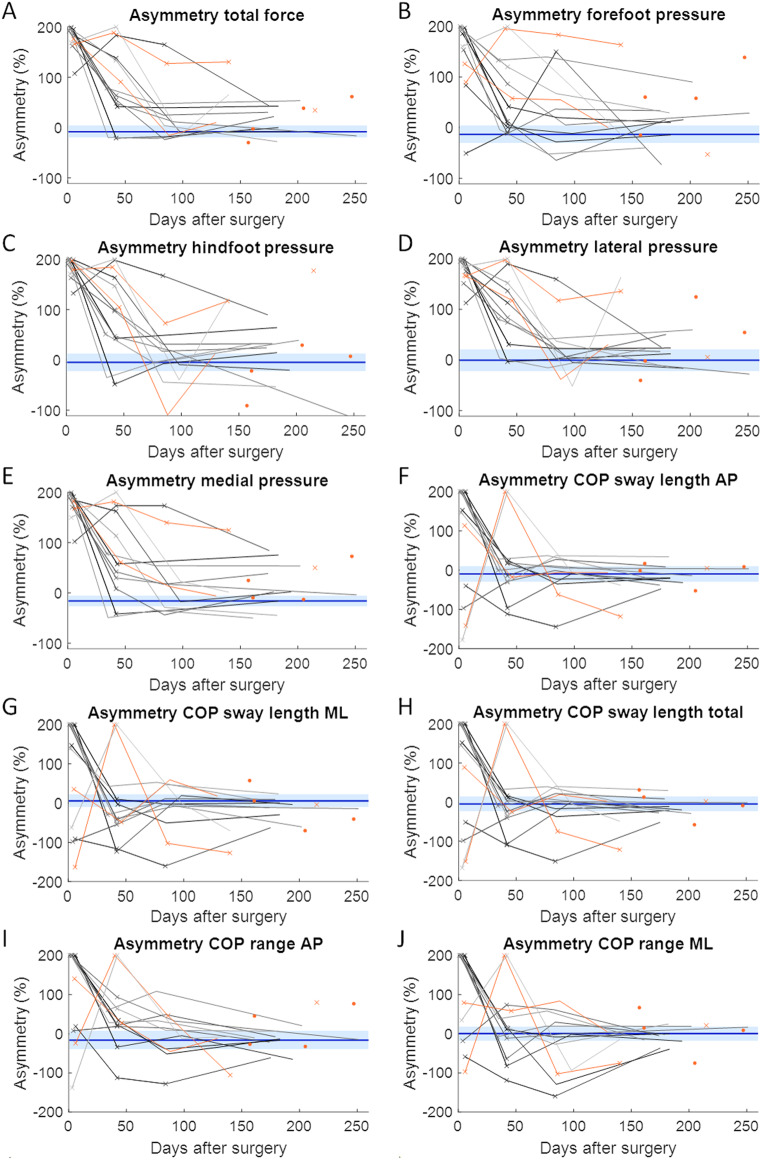
Asymmetry of weight and plantar pressure distribution parameters. A value of zero indicates perfect symmetry. The horizontal blue solid line and the light blue shaded area indicate the average and the 95% confidence intervals of the control group, respectively. Each longitudinally assessed patient is represented by a different line. The datapoints marked by a cross indicate that the patient performed the assessment with crutches. The datapoints and lines in orange present the patients experiencing nonunion. AP = anteroposterior; a.u. = arbitrary units; BW = body weight; COP = centre of pressure; ML = mediolateral.

The demographics of the patients with union were not significantly different from those of the age-matched controls or those of patients without union (Table 1). On average, measurements were obtained 4, 41, 86, and 176 days after surgery from the longitudinally assessed patients. Repeated-measures ANOVA revealed that all average weight and pressure distribution parameters improved during healing in patients with union, except for COP parameters in the AP direction and the total COP path length (Fig. 3). All asymmetry parameters improved over time (Fig. 4). Post hoc tests revealed that most improvements occurred during the first three months. Between the third (at three months) and fourth (at six months) visits, no significant changes were found (Table 2).

**Table 1:** Demographics of the patients and age-matched controls.

	Healthy controls	Patients with union	Patients with nonunion	*P* value controls vs. patients with union	*P* value patients with union vs. patients with nonunion
N (%female)	11 (73%)	11 (64%)	7 (29%)		
Age (years)	60.2 ± 11.2	58.9 ± 10.8	52.0 ± 22.0	0.774	0.467
Weight (kg)	74.9 ± 11.2	76.6 ± 13.9	82.3 ± 15.0	0.751	0.384
Height (m)	1.72 ± 0.07	1.73 ± 0.09	1.80 ± 0.09	0.748	0.217
Fracture site (proximal tibia/tibial shaft/malleolus)		5/2/4	1/6/0		

**Table 2:** Results of repeated-measures ANOVA only for patients with union (*n* = 11). Significant values are presented in bold.

	Repeated-measures ANOVA	Post hoc tests with Holm correction					
Parameter	P value (effect size)	T1-T2	T1-T3	T1-T4	T2-T3	T2-T4	T3-T4
Total force	**< 0.001 (0.72)**	**< 0.001**	**< 0.001**	**< 0.001**	**0.002**	**0.014**	0.358
Forefoot pressure	**< 0.001 (0.63)**	**0.016**	**< 0.001**	**< 0.001**	**0.016**	**0.009**	0.631
Hindfoot pressure	**< 0.001 (0.60)**	**0.006**	**< 0.001**	**< 0.001**	**0.016**	0.215	0.215
Lateral pressure	**< 0.001 (0.67)**	**0.007**	**< 0.001**	**< 0.001**	**0.003**	**0.012**	0.432
Medial pressure	**< 0.001 (0.69)**	**< 0.001**	**< 0.001**	**< 0.001**	**0.013**	0.112	0.281
COP sway path length, AP*	0.959 (0.00)						
COP sway path length, ML	**0.046 (0.23)**	0.070	0.127	0.140	1.000	1.000	1.000
COP sway path length, total*	0.815 (0.01)						
COP sway range, AP	0.165 (0.15)						
COP sway range, ML	**0.010 (0.31)**	**0.020**	**0.020**	0.114	1.000	1.000	1.000
Asymmetry, total force	**< 0.001 (0.73)**	**< 0.001**	**< 0.001**	**< 0.001**	**0.016**	**0.016**	0.991
Asymmetry, forefoot pressure*	**< 0.001 (0.56)**	**0.002**	**< 0.001**	**< 0.001**	0.743	0.283	0.743
Asymmetry, hindfoot pressure	**< 0.001 (0.66)**	**0.003**	**< 0.001**	**< 0.001**	**0.027**	**0.029**	0.847
Asymmetry, lateral pressure	**< 0.001 (0.69)**	**0.003**	**< 0.001**	**< 0.001**	**0.006**	**0.011**	0.722
Asymmetry, medial pressure*	**< 0.001 (0.69)**	**< 0.001**	**< 0.001**	**< 0.001**	0.063	0.063	0.864
Asymmetry, COP sway path length, AP*	**0.030 (0.35)**	**0.021**	**0.013**	**0.009**	1.000	1.000	1.000
Asymmetry, COP sway path length, ML*	**0.009 (0.42)**	**0.003**	**0.005**	**0.003**	1.000	1.000	1.000
Asymmetry, COP sway path length, total*	**0.028 (0.35)**	**0.014**	**0.014**	**0.009**	1.000	1.000	1.000
Asymmetry, COP sway range, AP*	**0.005 (0.50)**	**0.012**	**< 0.001**	**< 0.001**	0.526	0.279	0.557
Asymmetry, COP sway range, ML	**< 0.001 (0.55)**	**< 0.001**	**< 0.001**	**< 0.001**	1.000	1.000	1.000

*Sphericity was violated and corrected for with the Greenhouse‒Geisser method; T = measurement timepoint.

Approximately six months after surgery, among the patients with union, the average values of the total force, hindfoot pressure, medial pressure, all COP sway path length parameters, and COP sway range in the ML direction were still significantly different from those in the age-matched controls. Regarding asymmetry parameters, the total force, lateral pressure, medial pressure and COP sway path length in the ML direction were significantly different between controls and patients with union (Table 3). The largest effect size was found for the total force. No significant differences in weight or pressure distribution parameters were found between patients with or without fracture union at approximately six months after surgery (Table 3).

**Table 3:** Results of comparisons between controls and patients with union, as well as patients with and without fracture union six months after surgery. Significant values are presented in bold.

	Comparison between controls and patients with union	Comparison between patients with and without union
Parameter	P value (effect size)	P value (effect size)
Total force	**0.002 (1.50)**	0.605 (0.26)
Forefoot pressure	0.413* (0.21)	0.553 (0.43)
Hindfoot pressure	**0.030 (0.97)**	0.675 (0.21)
Lateral pressure	0.092 (0.74)	0.663 (0.22)
Medial pressure	**0.007 (1.24)**	0.355 (0.46)
COP sway path length, AP	0.037* (-0.52)	0.596* (0.17)
COP sway path length, ML	0.006* (-0.67)	1.000* (-0.01)
COP sway path length, total	0.016* (-0.59)	0.536* (0.20)
COP sway range, AP	0.379* (-0.23)	1.000* (-0.01)
COP sway range, ML	0.037* (-0.52)	0.930* (0.04)
Asymmetry, total force	**0.011 (-1.17)**	0.414 (-0.41)
Asymmetry, forefoot pressure	0.140 (-0.64)	0.155 (-0.72)
Asymmetry, hindfoot pressure	0.081 (-0.77)	0.743 (-0.16)
Asymmetry, lateral pressure	0.027* (-0.55)	0.724* (-0.12)
Asymmetry, medial pressure	**0.024 (-1.01)**	0.354 (-0.46)
Asymmetry, COP sway path length, AP	0.129 (0.66)	0.596* (-0.17)
Asymmetry, COP sway path length, ML	**0.019 (1.06)**	0.864 (0.08)
Asymmetry, COP sway path length, total	0.057 (0.84)	0.868 (0.08)
Asymmetry, COP sway range, AP	0.282 (0.46)	0.363 (-0.45)
Asymmetry, COP sway range, ML	0.210 (0.54)	0.917 (-0.05)

*Nonnormally distributed data analysed by Mann‒Whitney U test.

## Discussion

The present study analysed the weight and plantar pressure distributions throughout the first six months after tibial or malleolar fractures. Almost all average parameters (except for some of the COP-related parameters) and all asymmetry parameters improved throughout the first six months in patients who achieved union. The greatest improvements occurred within the first three months. However, at six months, more than half of the parameters were still significantly different from those of age-matched controls. The weight and plantar pressure distributions during standing did not differ between patients who did and did not achieve fracture union at approximately six months after surgery. Therefore, stance analysis does not have potential to be used as an early predicting marker of potential healing problems. The information about weight and pressure distribution can, however, be used to personalize the (physical) therapy plan.

To our knowledge, this is the first longitudinal study that provides data on weight and pressure distributions while standing after tibial or malleolar fractures. After a hip fracture, the amount of postural sway is related to preinjury mobility and postoperative thigh oedema^23,24^. Approximately one year after a lower leg fracture, the plantar pressure is located more lateral during walking^2^. This was not observed during standing in our study; the lateral pressure of patients was not significantly different from that of the healthy controls and even tended to be slightly lower than that of the controls at six months. The results showed that the medial pressure was significantly lower, and the asymmetry of the medial and lateral pressure in patients was significantly greater than that in the controls (Table 3). These findings indicate that there are changes in plantar pressure in the mediolateral direction that are still present six months after surgery.

Two of the longitudinally assessed patients were diagnosed with nonunion. Only one of them performed clearly worse than the other patients during the last two measurements. This patient had type 2 diabetes and smoked, which are known risk factors for nonunion^22^. The performance of the second longitudinally assessed nonunion patient did not deviate from that of the other patients; however, this patient might have repeatedly put too much weight on the implant, which led to screw breakage. The instability of the fracture after the screws broke could have prevented the fracture from healing. The last measurements of these longitudinal patients who developed nonunion were added to those of the group of patients with nonunion who were only assessed once approximately six months after surgery for analysis.

The weight and pressure distributions of patients with nonunion of a tibial fracture did not differ from those of patients with union during the standing task. Four of the seven nonunion patients had undergone treatment with a tibial nail. During simple static movements, such as standing, it is likely that all of the load passed through the nail and that the patients would not have been bothered much by their nonunion. Dynamic movements might have more potential to distinguish between patients with and without fracture union.

One of the longitudinally assessed patients who achieved union performed slightly worse than the other patients who achieved union. This patient still used crutches three months after surgery and put clearly less load on the injured side than the other patients. Reconstruction of the joint surface was not optimal in this patient, and the joint surface had irregularities of 5 mm. This could be the reason why this patient improved more slowly than the other patients. Stance analysis might not be sensitive enough to detect all healing problems, but it might be able to identify certain complications throughout the healing process.

Symmetrical weight bearing should be pursued at the end of the treatment since it is most stable from a biomechanical point of view. This means that the probability of balance recovery is equal in each direction. When weight is asymmetrically distributed, stability decreases^16,25,26^. Therefore, it is important that patients who experience a lower leg fracture recover symmetrical weight bearing. However, the results of our study indicate that six months after surgery, more than half of the parameters were not comparable to those of age-matched controls. It could be that it takes longer to return to control-like values or that the patients do not notice that their weight and pressure distributions are not symmetrical after several weeks of partial weight bearing. Similar results have been observed for gait, with multiple pressure-related gait parameters not reaching the levels in healthy controls several years after a lower leg fracture^9,10^.

Weight and pressure distributions are likely to be influenced by the amount of pain. In patients with low back pain, the COP sway length was greater than that in healthy controls^27,28^. In addition, patients with knee osteoarthritis also had more postural sway^29,30^. In low back pain patients, postural sway was reduced after manual pain relief interventions^31^, but pain medication did not affect postural sway in patients with knee osteoarthritis^32^. The pain intensity was also related to the amount of sway in patients with nonspecific neck pain^33^. Therefore, the amount of pain and painkiller intake could have affected our results.

All longitudinally assessed patients used crutches during at least one measurement. Although the use of crutches will affect the amount of weight placed on the injured side, it is likely that the amount of pain has a greater influence on how much weight is placed on the injured side compared to the use of crutches. Nonetheless, the use of crutches could have an effect on how pressure is distributed underneath the feet. It is known that crutch height does not affect COP measures during standing^34^. However, because the crutches are extra contact points, the base of support is larger, and patients can have larger sway movements without losing their balance. Our results clearly show that the COP sway path length and COP range parameters were greater when crutches were used than when they were not used. Therefore, COP measures are not suitable for monitoring improvements after lower leg fractures while standing as long as patients are still using crutches.

### Study limitations

A limitation of this study is the small sample size. Larger studies are needed to confirm the present findings and the benefits for routine clinical use. Additionally, the longitudinally assessed patients had different types of lower leg fractures. Malleolar fractures on average seem to heal faster compared to tibial fractures. However, because of the six weeks of partial weight bearing prescribed after fixation of a malleolar fracture in the present study, the difference in healing times between tibial and malleolar fractures is likely relatively small. Nonetheless, it could be the case that the fracture type influences weight and pressure distribution, although this appears not to be the case when visually inspecting our data. The same applies for the type of implant; both nails and plates were used for fracture fixation. Because of the small number of patients, the effects of these differences could not be analysed.

### Implementation in clinical practice

Stance analysis with either wearable insoles or pressure plates is relatively easy to implement in clinical practice. Patients can easily perform the standing task (with or without crutches), which only takes 10 s. Most hardware, such as pressure plates or pressure-sensing insoles, has associated software that automatically reports data collected directly after the measurement, which can be used by healthcare professionals to monitor progress between clinical examinations.

## Conclusion

Improvements in weight and plantar pressure distribution throughout the healing phase could be measured with a simple standing task. However, COP measures were not suitable for monitoring changes when patients used crutches. Six months after fracture, several parameters were still significantly different between patients and age-matched controls, indicating that patients had not yet returned to a normal stance. It was not possible to discriminate between patients with and without fracture union based on weight and plantar pressure distribution while standing. However, this method has the potential to detect certain abnormalities throughout the healing process earlier.

## Data Availability

Data are available upon reasonable request. Please contact the corresponding author.
